# A norm knockout method on indirect reciprocity to reveal indispensable norms

**DOI:** 10.1038/srep44146

**Published:** 2017-03-09

**Authors:** Hitoshi Yamamoto, Isamu Okada, Satoshi Uchida, Tatsuya Sasaki

**Affiliations:** 1 Rissho University, Department of Business Administration, Tokyo, 141-8602, Japan; 2 Soka University, Department of Business Administration, Tokyo, 192-8577, Japan; 3 RINRI Institute, Research Center for Ethiculture Studies, Tokyo, 102-8561, Japan; 4 University of Vienna, Faculty of Mathematics, Vienna, 1090, Austria

## Abstract

Although various norms for reciprocity-based cooperation have been suggested that are evolutionarily stable against invasion from free riders, the process of alternation of norms and the role of diversified norms remain unclear in the evolution of cooperation. We clarify the co-evolutionary dynamics of norms and cooperation in indirect reciprocity and also identify the indispensable norms for the evolution of cooperation. Inspired by the gene knockout method, a genetic engineering technique, we developed the norm knockout method and clarified the norms necessary for the establishment of cooperation. The results of numerical investigations revealed that the majority of norms gradually transitioned to tolerant norms after defectors are eliminated by strict norms. Furthermore, no cooperation emerges when specific norms that are intolerant to defectors are knocked out.

Reciprocity is a fundamental mechanism that underlies all cooperative societies. Theoretically it is well known that direct reciprocity, typified by the ‘‘I’ll help you if you help me” attitude, promotes cooperative regimes^1^,^2^. However, in recent societies that have high relational mobility, indirect reciprocity such as ‘‘I’ll help you and somebody else will help me” plays a more important role in promoting cooperation. Indirect reciprocity has therefore been the focus of much research in the interdisciplinary fields in recent decades^3^,^4^,^5^,^6^,^7^.

Many theoretical studies on indirect reciprocity have explored norms that become evolutionarily stable against defection and the invasion of free riders, and several typical norms have been proposed^8^,^9^,^10^,^11^. These approaches have clarified the robust norms that can maintain the cooperative regime. The norms in the studies on the indirect reciprocity are regarded as assessment rules that label the other’s action as either Good or Bad. They include tolerant norms that assess cooperative behaviors toward defectors as good^4^ and strict norms that assess such behaviors as bad^12^. Other theoretical studies analysing the global dynamics of norms assume that at most a few robust norms are shared in the population^13^,^14^,^15^.

Their approaches have clarified the robustness of the norms against invasion of other norms including free riders when the norms are acceptable in the population. However, little is known about a process by which gradual changes toward cooperation occur as new norms emerge and compete, which is to say, the co-evolutionary process of norm-diversity and cooperation. A study on the indirect reciprocity has dealt with co-existing different norms and has analysed their frequencies in the population as a consequence of a dynamical process^16^. In the study, each individual keeps a private image of everyone else and errors of perception and implementation are included in a limited strategy space. Although they have considered some action rules and assessment rules, all possible norms in indirect reciprocity have not been studied all-together. How cooperation evolves cannot be fully understood unless the evolution of norms is also considered.

It is thus a challenging task to theoretically understand how cooperation can be formed even under a collection of norms in a social system. How is the co-existence of cooperation and diversity possible at all? Are there any indispensable norms needed to facilitate the evolution of cooperation in the melting pot of norms, even though some norms never become dominant? Do norms that could be accepted as a result of the co-evolutionary process have common aspects? These questions can be addressed only if all possible norms are considered, and the combination of norms governing a group can evolve.

Here we explore the dynamics of co-evolution of cooperation by using different social norms. The process of the evolution of norms has a transition from stricter to tolerant norms. Additionally, we find a set of norms that seem not to have an impact on promoting cooperation, but are fundamental to allow a transition to a cooperative regime from a defective regime.

## Results

**Figure 1:**
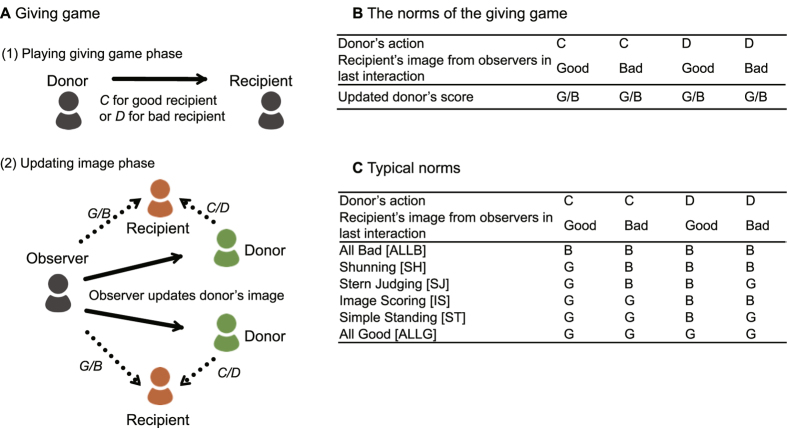
The norms of cooperation and simulation framework. (**A**) (1) If the donor’s image of a recipient is Good, the donor gives the recipient something with personal cost *c* and the recipient receives benefit *b*. Nothing happens otherwise. (2) In the Updating image phase, the observer updates the evaluation to the donor on the basis of the donor’s action (Cooperation [C]/Defection [D]) and the observer’s evaluation (Good [G]/Bad [B]) of the recipient. (**B**) Each agent adopts an evaluation rule of the donor that depends on the donor’s action and the recipient’s image. This combination of Good/Bad is the norm held by the agent. There are a total of 2^4^ = 16 possible norms. In this phase, each agent evaluates and updates its image to all donors. (**C**) Typical norms can be expressed in the manner shown in this table. Typical norms include Shunning [SH] = GBBB, Stern Judging [SJ] = GBBG, Image Scoring [IS] = GGBB, and Simple Standing [ST] = GGBG. SH is a strict norm where any action for a Bad recipient is assessed as Bad. ST is a tolerant norm where any action for a Bad recipient is assessed as Good. SJ is an intermediately strict norm where cooperation for a Bad recipient is assessed as Bad while defection is Good. In contrast, IS does not use an image to recipient but uses only donor’s action. If the donor’s previous action is C, then IS evaluates the donor as Good, otherwise IS evaluates the donor as Bad.

**Figure 2:**
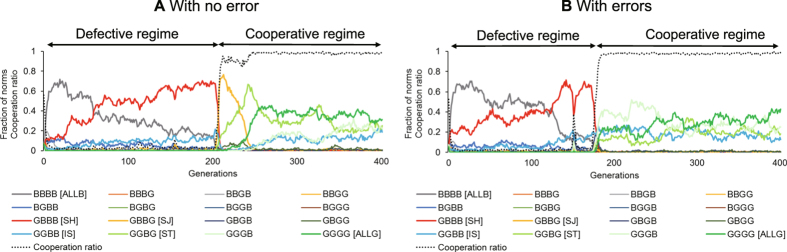
Time series of typical simulation runs with all norms. With no error (left panel) and with errors (right panel). (**A**) The average frequencies of 16 norms and the cooperation of the overall society. The black dotted line is the cooperation ratio. Parameters: *b* = 5, *c* = 1, *N* = 500, *R* = 500, *G* = 1000, *p* = 0, *q* = 0. When SH and ALLB coexist, cooperation does not emerge. When ALLB is completely driven out by SH, SJ invades and the cooperation ratio abruptly rises. At the same time, SH is driven out by SJ. After cooperation is completely achieved, SJ permits the invasion of ST, and also coexists with other tolerant norms (IS, ST, GGGB, and ALLG). Finally, strategies whose norm is expressed as GG** (in other words, norms that constantly cooperate if cooperation has been selected in the past by the recipient) coexist. In (**B**), both errors in perception and implementation were introduced, and simulation similar to a was run (*b* = 5, *c* = 1, *N* = 500, *R* = 500, *G* = 1000, *p* = 0.001, *q* = 0.001). As in (**A**), when SH and ALLB coexist, cooperation does not emerge. However, cooperation is achieved without going through SJ.

**Figure 3:**
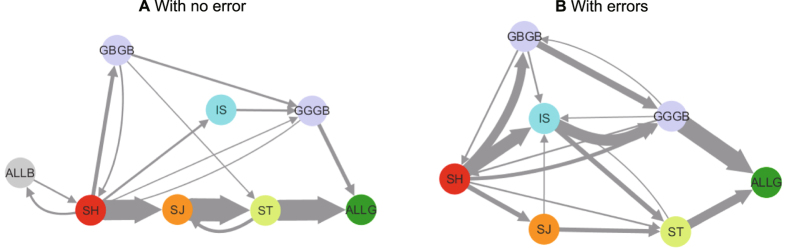
The alternation patterns of the majority of norms with 50 replications. With no error (left panel) and with errors (right panel). (**A**) The panel shows the transition of norms with the greatest populations in a round of 20 generations, before the cooperation ratio exceeds 0.8, and 100 generations, after the cooperation ratio exceeds 0.8 (for a total of 120 generations). For the sake of visibility, in a replication, we stop calculation when ALLG becomes the majority norm. This is because in a state in which tolerant norms coexist, the norms with the greatest population frequently change place. The thickness of the arrows corresponds to the number of times alternation of norms occurred. (See the Supplementary Information for details.) The alternation of norms SH → SJ → ST → ALLG was observed to be stable. In (**B**), both errors in perception and implementation were introduced, and simulations similar to a were run (*b* = 5, *c* = 1, *N* = 500, *R* = 500, *G* = 1000, *p* = 0.001, *q* = 0.001). As shown in (**B**), the transition of majority norms is not distinct compared to the times when there were no errors.

Agent-based simulations^17^,^18^ are an optimal tool to tackle the challenge outlined above. See Methods for the details of our agent-based model described by the ODD protocol^19^. Using an evolutionary game theoretical framework and constructing an interaction model based on players’ private rules and local information, we model a giving game to elucidate the dynamics of the evolution of cooperation amid the coexistence of diverse norms (Fig. 1). We conducted numerical simulations of all 16 possible norm combinations that could react to the four combinations of assessment criteria to clarify the dynamics of the evolution of cooperation from the melting pot of diverse norms. Figure 2A shows time-series graphs of each norm’s population and cooperation ratio. As shown, the majority undergo an alternation from strict to tolerant norms, mostly in the order of SH → SJ → ST. Figure 3A shows the transition in the norm with the greatest population ratio. In many cases, the majority transitioned from the state where strict SH^11^ was the majority to SJ^5^,^12^. Afterwards, the majority norm changed to tolerant ST^4^,^20^,^21^ and ALLG. In contrast, as shown in Figs 2B and 3B, in an environment with errors, alternation from strict norms to tolerant norms was observed. However, the likelihood of going through SJ decreased. Alternation paths through IS^22^,^23^, which could not be seen in an environment without errors^24^, increased. It is important to note here that similar paths toward cooperation are observed when only ALLB-individuals are initially assumed. New norms are created during the evolutionary process at the same time cooperation evolves. This indicates that cooperation and diversity of norms jointly evolve in the model.

Why does the alternation of norms emerge? For one thing, in states in which defection is dominant, ALLB (BBBB) and SH (GBBB) coexist and jointly form the majority. However, BGBB and IS (GGBB) continue to exist as the minority. The characteristic of these groups is having the evaluation rule of **BB. Evaluation rule **BB assesses donors that took D as B, regardless of the evaluation of the recipient. In states in which defection is dominant, those who adopt **BB strategies consider many partners as B. As a result, cooperation does not occur for the most part. The ALLB and SH norms thus survive because they do not lower their own cost. On the other hand, after cooperation is achieved, ALLG (GGGG), ST (GGBG), IS (GGBB), and GGGB coexist. The common characteristic of these norms is having the evaluation rule of GG**. Thus, reciprocally cooperating norms survive. Because SJ (GBBG), which becomes the majority temporarily when the cooperation ratio rises in an environment without errors, does not belong to either group, it cannot stably exist. Also, it is rare that SJ makes up the majority temporarily in an environment with errors. Meanwhile, because IS belongs to both norm groups with **BB and GG**, IS can constantly exist.

We discover several norms that are indispensable to the evolution of cooperation. Reputation-based cooperation cannot emerge without indispensable norms. To elucidate indispensable norms for the evolution of cooperation, we propose a novel analysis using the norm knockout method. This method enables us to determine which norms are indispensable for the evolution of cooperation. The norm knockout method is inspired by the targeted gene knockout technique used in genetic engineering^25^. Gene knockout, a genetic technique in which one of an organism’s genes is made inoperative, is used to research genes whose sequences are known but whose functions are not well-understood. Researchers infer the gene’s function from differences between the knockout animal and a normal animal. For simulating evolution, we utilized a method that removed only one particular norm from the population to understand whether that norm is an indispensable one that plays a critical role in the evolution of cooperation.

**Figure 4:**
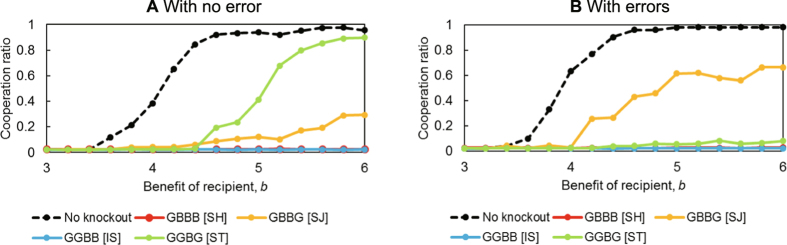
The cooperation ratio in the norm knockout method. Each graph shows the average cooperation ratio of 50 replications when a typical norm is knocked out. The basic parameter set is *c* = 1, *N* = 500, *R* = 500, *G* = 1000. To confirm the effects of errors in perception and errors in implementation, two simulations with and without error were executed. See the Supplementary Information for knockout analysis of all norms. (**A**) The case when errors in perception (*p*) and errors in implementation (*q*) are 0. When SH or IS is knocked out, cooperation does not evolve at all. Also, when SJ, which becomes the majority for only a brief round during the process of alternation, is knocked out, cooperation evolves to the extent of only 30 percent, even when *b* is large. Furthermore, when ST is knocked out, the range in which cooperation is achieved becomes narrow. Only when *b* is sufficiently large can cooperation evolve. (**B**) The case where *p* = *q* = 0.001. The indispensable norm is ST in addition to SH and IS. Conversely, when SJ is knocked out, cooperation evolves when *b* is sufficiently large in the same manner as ST in (**A**).

Figure 4 shows the cooperation ratio when a particular norm is knocked out. Regardless of whether there is an error, if SH or IS is knocked out, cooperation does not evolve at all. We define indispensable norms in the evolution of cooperation as the norms that, when knocked out, have an average cooperation ratio of less than 0.1 after 1,000 generations. In an environment with no errors, SH and IS are indispensable norms. In an environment with errors, SH, IS, and ST are indispensable norms.

**Figure 5:**
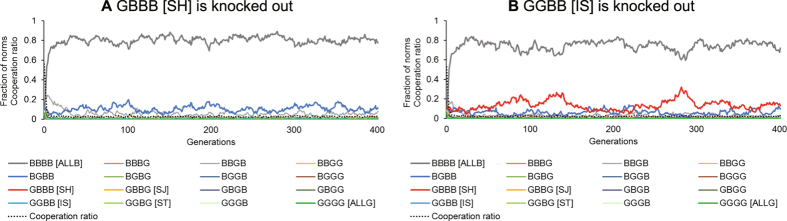
Time series of typical simulation runs in norm knockout method. The parameters are *b* = 5, *c* = 1, *N* = 500, *R* = 500, *p* = *q* = 0. (**A**) When SH is knocked out, the strategy to eliminate ALLB does not exist. (**B**) When IS is knocked out, SH exists only in a small population and cannot gain superiority over ALLB.

When an indispensable norm is knocked out, cooperation does not evolve. When cooperation evolves, alternation from strict norms to tolerant norms was observed, as shown in Figs 2 and 3. To analyse whether alternation also occurs when a norm is knocked out, the population ratio of norms when typical norms are knocked out is displayed as time-series graphs (see Fig. 5). Figure 5 shows the results in the cases where SH or IS were knocked out. We discovered that the first condition for the necessary process when cooperation evolves is whether SH can antagonize ALLB. No norm that resists the invasion of ALLB appears in a society in which SH does not exist. Also, in a society in which IS does not exist, SH cannot antagonize ALLB. We found that IS is a norm indispensable for SH to resist ALLB.

## Discussion

Our model offered two major findings on the evolution of cooperation on indirect reciprocity. On the one hand, the most essential contribution is the discovery of indispensable norms by the norm knockout method. By using the norm knockout method, we were able to elucidate the existence of norms indispensable for the evolution of cooperation from a melting pot of norms. Regardless of the existence of errors, SH and IS were indispensable norms. In addition, in an environment with errors, ST is an indispensable norm. Interestingly, SH and IS are reconciled to the minorities after the cooperative regime emerges while they temporarily become major norms in the process of dynamics. We call such minority norms required for the evolution of cooperation “unsung hero norms”. The results clearly illustrate the two roles of norms: one to catalyse a cooperative regime and the other to maintain the regime. Norms having the GG** for the evaluation rule play the latter role.

On the other hand, we discovered alternation of norms. Recent analysis of evolutionary stability against the invasion of free riders could identify neither superiority among norms nor the process on the path to cooperation. Among studies on indirect reciprocity, ours is the first exhaustive theoretical analysis on all possible norms, although several studies have addressed the comparison of two types of reciprocal norms^14^,^15^,^26^. Others analyse the alternation of norms in direct reciprocity^27^,^28^,^29^. We find the alternation of the norms and also discover the indispensable norms that are required to foster indirect reciprocity.

An empirical study^30^ supports the co-existence of various norms in the cooperative regime and indicates that the ST norm plays a more important role in human cooperation than SJ, which is consistent with our simulation. This is because we show that the SJ norm cannot survive in the cooperative regime, while the ST one can. Our approach may provide deep insight on the evolution of cooperation because several norms absolutely play an essential role in order to evolve cooperation even though, on the surface, it seems as though they are not directly leading to the evolution of the cooperation.

The present work considers a single action rule (cooperate with Good, defect with Bad) to stress the role of multiple assessment rules. However, the other papers stress the role of multiple co-existing action rules^13^,^31^,^32^,^33^. Integrating the multiple assessment and action rules may be a useful extension of this paper. We analyse what happens when one norm is absent from the population; however, we have not analysed all the indispensable combinations of norms yet. Extending the norm knockout method to combinations of norms may also be a useful extension of this paper.

## Methods

In this section, we describe the details of our agent-based model that uses the norm knockout method. The following model description follows the ODD protocol^19^.

### Purpose

The aim of the model is to understand the dynamics of norms during the evolution of cooperation, and to find indispensable norms without which cooperative societies could never emerge. In particular, we reveal the effect of these indispensable norms on indirect reciprocity using a new methodology we call the “norm knockout method”. We utilize the giving game framework^34^ for simulation.

### Entities, state variables, and scales

The entities in the model are agents who play as donor and recipient in the giving game with no spatial structures. The donor chooses cooperation or defection with a recipient using an image that the donor has to the recipient. An image is either Good or Bad. If a donor’s image to a recipient is Good, the donor cooperates with the recipient. If the image is Bad, the donor defects. The group size of the model is *N*. Each agent has it’s own norm and a list of images to other agents. The agent also has a probability of errors and a payoff of the game.

The norm of an agent is denoted as one of four possible “assessment combinations”, and there are two possible “alleles (G/B)” at the “locus” for each of the four assessment combinations. The first locus of the gene represents an assessment rule to an agent who cooperates with a Good recipient. The second locus represents an assessment rule to an agent who cooperates with a Bad recipient. The third locus represents an assessment rule to an agent who defects with a Good recipient. The fourth locus represents an assessment rule to an agent who defects with a Bad recipient. Incidentally, all agents evaluate themselves as Good. For instance, ALLG always assesses others as Good, and thus, its “genotype” is GGGG using the above mentioned definition of the four loci. Similarly, ALLB is described as BBBB, IS as GGBB, ST as GGBG, and SJ as GBBG.

Each agent has two different types of errors: one, the probability that the agent’s updating of its evaluation of others, Good/Bad, is inverted (errors in perception), described as *p*, and two, the probability to perform an action differently from the one prescribed by its action rule (errors in implementation), described as *q*. The evolution process of norms involves adopting a genetic algorithm^35^. State variables and initialization in the simulation are shown in Table 1.

### Process overview and scheduling

Our simulation runs throughout *G* generations. A generation consists of *R* rounds. The agents play the giving game *R* times as donor in each generation. At the end of a generation, they evolve their own norms using accumulated payoffs that are obtained in the generation. One round has two phases: (A) a phase to play giving games and (B) a phase to update images. After all agents play giving games in phase (A), all agents update their images in phase (B). In phase (A), each agent becomes a donor and each donor randomly chooses a recipient from *N* − 1 players excluding itself. The donor chooses whether to give benefits to the recipient or not. At that time, the action of the donor is inverted with the probability *q*. The donor who cooperates pays cost *c* and the recipient receives benefit *b (b* > *c* > 0). In phase (B), each agent (set to *i*) evaluates and updates an image to the other agent (set to *j*). The new image to *j* from *i*’s viewpoint depends on *j*’s action (C/D) as a donor in the last round and depends on the image to *k* from the viewpoint of *i* (G/B), where *k* was a recipient of *j* in the last round. At that time, the image to *j* is inverted with the probability *p*. In the first round in a generation, *j*’s action (C/D) is regarded as random.

After *R* rounds of the giving game are played in every generation, agents evolve their norm. The evolution process of norms involves adopting the genetic algorithm^35^. Because each locus of norm has an independent meaning for assessment to others, the adaptive process should contain a combination of these elements rather than a string of norms. We have modeled a process of updating norms not as a string of norms but rather as four different assessment rules, which enables the norms to be interpreted as different situations depending on the norm genotype. The first, second, third, and fourth loci represent the assessment for pro-social behavior, tolerant behavior, anti-social behavior, and justified defection (punishment), respectively. Each agent randomly selects two agents from *N* agents (including itself) to become its parents. For choosing parents, we adopt a roulette selection method. This roulette selection sets a probability distribution of all agents as Π_*i*_ = (*U*_*i*_ − *U*_*min*_)^2^/∑_*j*_(*U*_*j*_ − *U*_*min*_)^2^, where *U*_*i*_ denotes the agent *i*’s accumulated payoff in a generation given by *U*_*i*_ = *bW* − *cV*, with *W* being the number of donations *i* received in the generation and *V* the number of donations *i* gave. *U*_*min*_ means a minimum value of the accumulated payoffs among all. Finally, each agent updates its norm using a uniform crossover technique. With mutation rate *m*, each locus is inverted for maintaining the diversity of the norm space.

### Design concepts

#### Basic principles

An agent-based simulation is utilized to study indirect reciprocity. We explore how different combinations of norms interact to produce an evolutionary progression towards cooperation.

#### Emergence

A cooperative regime in the situation of social dilemma emerges from interactions among agents who have various social norms.

#### Adaptation

The agents of the model play the giving game using their images to others. The agents update their images to others using their norms every round. They evolve their own norms using accumulated payoffs that are obtained in the generation. A norm that can obtain a higher payoff can increase the population through the generation.

#### Objectives

The objective of all agents is to maximize their own payoff. To maximize payoff, they change their own norm at the end of each generation.

#### Learning

The agents change their norms in each generation using a genetic algorithm. The fitness of each agent is calculated from the accumulated payoff in the generation. To select the parents of the agent, the model utilizes a roulette selection method.

#### Interaction

The interaction between the agents is one to one interaction. The giving game consists of the donor and the recipient. There are no spatial structures in the society.

#### Stochasticity

The interaction between agents is a stochastic process because interaction partners are chosen randomly from the society. At the start of the simulation, each agent is randomly assigned a norm of all 16 norms.

#### Observation

Three indexes are used for observation: average cooperation ratio in the society, the transition of norms with the greatest populations, and population ratio of each norm.

### Initialization

At the start of simulation, the norm of each agent is chosen randomly from all 16 possible norm combinations. In the first round of each generation, the evaluation of all agents is initialized as Good^11^,^23^,^36^ and payoff of the agents is initialized as 0.

### Input data

After initialization, the model does not include any external inputs, i.e., the number of agents (*N*), error ratio (*p*, *q*), benefit (*b*), and cost (*c*) are constant.

### Submodels

#### The norm knockout method

The norm knockout method is implemented as follows. When we knock out a particular norm, that norm is removed in the first round of each generation. Concretely, if the norm of an agent evolves into a norm that is knocked out as a result of the adopting process, the norm of the agent is changed to one of the other 15 norms randomly. In other words, the norm that is knocked out will never exist at all in the society.

## Additional Information

**How to cite this article:** Yamamoto, H. *et al*. A norm knockout method on indirect reciprocity to reveal indispensable norms. *Sci. Rep.* **7**, 44146; doi: 10.1038/srep44146 (2017).

**Publisher's note:** Springer Nature remains neutral with regard to jurisdictional claims in published maps and institutional affiliations.

## Supplementary Material

Supplementary Information

## Figures and Tables

**Table 1:** State variables and initialization in the simulation.

Variable	Description	Type of variable	Initial value
Agent			
Norm	Norm of agent	16 types	chosen randomly
Image	Images to other agents	Binary (G/B)	G
Payoff	Accumulated payoff of the giving game	Real Number	0
*p*	Errors in perception	Constant	{0, 0.001}
*q*	Errors in implementation	Constant	{0, 0.001}
Environment			
*N*	Number of agents	Constant	500
*G*	Generations of simulation	Constant	1000
*R*	Times of playing giving game per generation	Constant	500
*b*	The benefit of giving game	Constant	[3.0, 6.0]
*c*	The cost of giving game	Constant	1
*m*	The mutation ratio	Constant	0.01

## References

[b1] Trivers R. L. The evolution of reciprocal altruism. Q. Rev. Biol. 46, 35–57 (1971).

[b2] Axelrod R. & Hamilton W. D. The evolution of cooperation. Science 211, 1390–1396 (1981). 7466396 10.1126/science.7466396

[b3] Alexander R. The Biology of Moral Systems (Aldine de Gruyter, New York, 1987).

[b4] Sugden R. The Economics of Rights, Cooperation and Welfare (Basil Blackwell, Oxford, 1986).

[b5] Kandori M. Social norms and community enforcement. Rev. Econ. Stud. 59, 63–80 (1992).

[b6] Wedekind C. & Milinski M. Cooperation through image scoring in humans. Science 288, 850–852 (2000). 10797005 10.1126/science.288.5467.850

[b7] Panchanathan K. & Boyd R. Indirect reciprocity can stabilize cooperation without the secondorder free rider problem. Nature 432, 499–502 (2004). 15565153 10.1038/nature02978

[b8] Ohtsuki H. & Iwasa Y. How should we define goodness? -reputation dynamics in indirect reciprocity. J. Theor. Biol. 231, 107–120 (2004). 15363933 10.1016/j.jtbi.2004.06.005

[b9] Nowak M. A. & Sigmund K. Evolution of indirect reciprocity. Nature 437, 1291–1298 (2005). 16251955 10.1038/nature04131

[b10] Ohtsuki H. & Iwasa Y. The leading eight: social norms that can maintain cooperation by indirect reciprocity. J. Theor. Biol. 239, 435–44 (2006). 16174521 10.1016/j.jtbi.2005.08.008

[b11] Takahashi N. & Mashima R. The importance of subjectivity in perceptual errors on the emergence of indirect reciprocity. J. Theor. Biol. 243, 418–36 (2006). 16904697 10.1016/j.jtbi.2006.05.014

[b12] Pacheco J. M., Santos F. C. & Chalub F. A. C. Stern-judging: A simple, successful norm which promotes cooperation under indirect reciprocity. PLoS Comput. Biol. 2, e178 (2006). 17196034 10.1371/journal.pcbi.0020178 PMC1761656

[b13] Ohtsuki H. & Iwasa Y. Global analyses of evolutionary dynamics and exhaustive search for social norms that maintain cooperation by reputation. J. Theor. Biol. 244, 518–531 (2007). 17030041 10.1016/j.jtbi.2006.08.018

[b14] Uchida S. & Sigmund K. The competition of assessment rules for indirect reciprocity. J. Theor. Biol. 263, 13–19 (2010). 19962390 10.1016/j.jtbi.2009.11.013

[b15] Uchida S. Effect of private information on indirect reciprocity. Phys. Rev. E 82, 036111 (2010). 10.1103/PhysRevE.82.036111 21230143

[b16] Brandt H. & Sigmund K. The logic of reprobation: Assessment and action rules for indirect reciprocation. J. Theor. Biol. 231, 475–486 (2004). 15488525 10.1016/j.jtbi.2004.06.032

[b17] Gilvert N. & Troitzsch K. Simulation for the Social Scientist (Open University Press, 1999).

[b18] Roberts G. Evolution of direct and indirect reciprocity. Proc. R. Soc. Lond. B 275, 173–179 (2008). 10.1098/rspb.2007.1134 PMC2596181 17971326

[b19] Grimm V. . The odd protocol: A review and first update. Ecol. Modell. 221, 2760–2768 (2010).

[b20] Leimar O. & Hammerstein P. Evolution of cooperation through indirect reciprocity. Proc. R. Soc. Lond. B 268, 745–753 (2001). 10.1098/rspb.2000.1573 PMC1088665 11321064

[b21] Panchanathan K. A tale of two defectors: the importance of standing for evolution of indirect reciprocity. J. Theor. Biol. 224, 115–126 (2003). 12900209 10.1016/s0022-5193(03)00154-1

[b22] Nowak M. A. & Sigmund K. Evolution of indirect reciprocity by image scoring. Nature 393, 573–577 (1998). 9634232 10.1038/31225

[b23] Nowak M. A. & Sigmund K. The dynamics of indirect reciprocity. J. Theor. Biol. 194, 561–574 (1998). 9790830 10.1006/jtbi.1998.0775

[b24] Lotem A., Fishman M. & Stone L. Evolution of cooperation between individuals. Nature 400, 226–227 (1999). 10.1038/22247 10421362

[b25] Strepp R., Scholz S., Kruse S., Speth V. & Reski R. Plant nuclear gene knockout reveals a role in plastid division for the homolog of the bacterial cell division protein ftsz, an ancestral tubulin. Proc. Natl. Acad. Sci. USA 95, 4368–4373 (1998). 9539743 10.1073/pnas.95.8.4368 PMC22495

[b26] Matsuo T., Jusup M. & Iwasa Y. The conflict of social norms may cause the collapse of cooperation: indirect reciprocity with opposing attitudes towards in-group favoritism. J. Theor. Biol. 346, 34–46 (2014). 24380777 10.1016/j.jtbi.2013.12.018

[b27] Lindgren K. Evolutionary phenomena in simple dynamics. In Langton C. G., Taylor C., Farmer J. D. & Rasmussen S. (eds) *Artificial Life II*, 295–312 (Addison-Wesley, Redwood City, CA, 1992).

[b28] Zagorsky B. M., Reiter J. G., Chatterjee K. & Nowak M. A. Forgiver triumphs in alternating Prisoner’s Dilemma. PLoS ONE 8, e80814 (2013). 24349017 10.1371/journal.pone.0080814 PMC3861238

[b29] Berg P. V. D. & Weissing F. J. The importance of mechanisms for the evolution of cooperation. Proc. R. Soc. Lond. B 282, 20151382 (2015). 10.1098/rspb.2015.1382 PMC4632633 26246554

[b30] Swakman V., Molleman L., Ule A. & Egas M. Reputation-based cooperation: Empirical evidence for behavioral strategies. Evol. Hum. Behav. 37, 230–235 (2016).

[b31] Brandt H. & Sigmund K. Indirect reciprocity, image scoring, and moral hazard. Proc. Natl. Acad. Sci. USA 102, 2666–2670 (2005). 15695589 10.1073/pnas.0407370102 PMC548983

[b32] Santos F. P., Santos F. C. & Pacheco J. M. Social norms of cooperation in small-scale societies. PLoS Comput. Biol. 12, e1004709 (2016). 26808261 10.1371/journal.pcbi.1004709 PMC4726523

[b33] Sasaki T., Okada I. & Nakai Y. The evolution of conditional moral assessment in indirect reciprocity. *Sci. Rep.* 7, 41870 (2017). 10.1038/srep41870 PMC5288800 28150808

[b34] Sigmund K. The calculus of selfishness (Princeton University Press, 2010).

[b35] Holland J. Adaptation in Natural and Artificial Systems (University of Michigan Press, 1975).

[b36] Ohtsuki H., Iwasa Y. & Nowak M. A. Reputation effects in public and private interactions. PLoS Comput. Biol. 11, e1004527 (2015). 26606239 10.1371/journal.pcbi.1004527 PMC4659694

