# Supplementary material for: A norm knockout method on indirect reciprocity to reveal indispensable norms
Source: Sci Rep. 2017 Mar 9;7:44146. doi: 10.1038/srep44146 (PMC5343449; doi:10.1038/srep44146)
Supplement: Supplementary Information [file srep44146-s1.pdf]

Supplementary Information

A norm knockout method on indirect reciprocity to reveal indispensable norms

Hitoshi Yamamoto[1*], Isamu Okada[2+], Satoshi Uchida[3+], and Tatsuya Sasaki[4+]

[1] Rissho University, Department of Business Administration, Tokyo, 141–8602, Japan

[2] Soka University, Department of Business Administration, Tokyo, 192–8577, Japan

[3] RINRI Institute, Research Center for Ethiculture Studies, Tokyo, 102–8561, Japan

[4] University of Vienna, Faculty of Mathematics, Vienna, 1090, Austria

[*]hitoshi@ris.ac.jp

[+]These authors contributed equally to this work

This file includes:

Supplementary Text S1

Supplementary Tables S1 – S6

Supplementary Figure S1

Text S1

The details of alternation of norms

We present the details of alternating the majority of norms, as shown in Fig. 3 (in the Main Text). Table S1 shows the alternation with no error and Table S2 shows with errors.

The population of each norm

We show the average population ratio at the 1,000th generation (50 replications). As shown in Table S3, persistent norms are able to be described as GG**.

The details of results of norm knockout method

We comprehensively explore the indispensable norms by knocking out each of the 16 norms. Table S4 shows the cooperation ratio in which each norm is knocked out. Without errors, SH and IS are the indispensable norms. If SJ or ST is knocked out, their standard deviations (S.D.) are larger than other cases. The reason for this is that to knock out SJ or ST produces two contrasting results: cooperation dominant or defection dominant. When indispensable norms are knocked out, no cooperative regime appears at all, and the standard deviation has a very small value.

The analysis of the alternation of norms after cooperative regime achieved.

In this section, we have analysed the transition of norms with the greatest populations after a cooperation ratio exceeds 0.9 until the end of generations. For the sake of understanding the mechanism of co-evolution of norms and cooperation, we have focused on the duration of regime changes from defection to cooperation in the main text. Therefore, we stopped the calculation of the transition of the majority norm when ALLG becomes the majority norm in Fig. 3. Intuitively, it seems impossible to transit from a majority of ALLG to a majority of any other norm; however, it is well-known that a state where everyone cooperates indiscriminately is easily replaced by a state where everyone refuses to cooperate.

To clarify whether ALLG is a stable state and what would happen if more generations were considered, we show the transition of norms with the greatest population after a cooperative regime is achieved. The results (Tables S5 and S6, Fig. S1) show that the cooperation regime is maintained robustly and tolerant norms such as ALLG, GGGB, and ST coexist. Although ALLG forms the majority of the population, the other norms including GGGB, ST (GGBG), and IS (GGBB) protect against invasion from defective norms.

Tables

Table S1. The alternation patterns of dominant norms with no error correspond to the left panel of Fig. 3 (in the Main Text). Each row shows the transition of norms with the greatest populations in a period of 20 generations, before the cooperation ratio exceeds 0.8, and 100 generations, after the cooperation ratio exceeds 0.8 (for a total of 120 generations). For the sake of visibility, we stop calculation when ALLG becomes the majority norm. Fifty replications are conducted. During this time, alternation in majority norms for a total of 156 times could be observed. A cooperative regime with a cooperation ratio exceeding 0.8 was achieved in 46 replications. For example, over 50 replications, the number of times the transition of greatest population followed (SH → SJ → ST → ALLG) was 31. Moreover, four (indicated by the dash) never had a cooperation ratio exceeding 0.8.

Transition pattern of dominant strategies	No.
SH → SJ → ST → ALLG	31
SH → SJ → ST → SJ → ST → ALLG	5
–	4
SH → GBGB → GGGB → ALLG	3
SH → GBGB → SH → SJ → ST → ALLG	2
SH → IS → GGGB → ALLG	2
SH → IS → GGGB → SH → ALLB → SH → ALLB → SH → ALLB	1
SH → GGGB → ALLG	1
SH → GBGB → ST → ALLG	1

Table S2. The alternation patterns of dominant norms with errors correspond to the right panel of Fig. 3 (in the Main Text). The setting of the table is the same as Table S1.

Transition pattern of dominant strategies	No.
SH → IS → GGGB → ALLG	13
SH → GBGB → GGGB → ALLG	8
SH → SJ → ST → ALLG	7
SH → GGGB → ALLG	6
SH → IS → ST → ALLG	5
SH → ST → ALLG	2
SH → GBGB → GGGB → SH → GBGB → GGGB → SH → IS → GGGB → ALLG	1
SH → IS → ST → IS → GGGB → ALLG	1
SH → GBGB → SH → IS → GGGB → ALLG	1
SH → IS → GGGB	1
SH → GBGB → IS → GGGB → GBGB → GGGB → ALLG	1
SH → SJ → IS → ST → ALLG	1
SH → GBGB → IS → GGGB → ALLG	1
SH → GBGB → SH → IS → ST → ALLG	1
SH → GBGB → GGGB → IS → GGGB → ALLG	1

Table S3. The average population ratio at the 1,000th generation (*b* = 5). Each column shows without/with errors. All cells are obtained by averaging the results of 50 replications. These results show the population of each norm in which all norms exist (i.e., the norm knockout method is not used). The second row shows the average cooperation ratio (C_{ratio}) and standard deviation at the 1,000th generation. Below the fourth row is shown the population of each norm and its standard deviation. The norms described as GG** can coexist stably while any norm that is not GG** can barely exist. SH, which is an indispensable norm, also cannot survive. IS, which is included in four persistent norms GG**, is the most in minority of the four.

	$p = q = 0$	$p = q = 0.001$
C_{ratio} (S.D.)	0.939 (0.187)	0.980 (0.006)
Norms	Population (S.D.)	Population (S.D.)
BBBB [ALLB]	0.015 (0.085)	0.000 (0.001)
BBBG	0.001 (0.002)	0.000 (0.000)
BBGB	0.001 (0.003)	0.000 (0.001)
BBGG	0.000 (0.001)	0.000 (0.001)
BGBB	0.003 (0.007)	0.002 (0.002)
BGBG	0.003 (0.003)	0.002 (0.002)
BGGB	0.004 (0.004)	0.004 (0.003)
BGGG	0.005 (0.004)	0.005 (0.003)
GBBB [SH]	0.026 (0.109)	0.002 (0.003)
GBBG [SJ]	0.009 (0.006)	0.005 (0.004)
GBGB	0.020 (0.013)	0.007 (0.005)
GBGG	0.024 (0.012)	0.012 (0.006)
GGBB [IS]	0.132 (0.043)	0.148 (0.040)
GGBG [ST]	0.165 (0.073)	0.201 (0.071)
GGGB	0.271 (0.093)	0.271 (0.079)
GGGG [ALLG]	0.322 (0.090)	0.341 (0.064)

Table S4. Analysis of norm knockout method for each of the 16 norms. The table shows the cooperation ratio at the 1,000th generation in which each of the 16 norms is knocked out (b = 5). Each value shows the average cooperation ratio from 50 replications and standard deviation. The cells in which the average cooperation ratio is less than 0.1 are shown in red. In this paper, we call these norms "indispensable norms". With no error, SH and IS are indispensable norms. With errors, these two plus ST are indispensable norms.

	$p = q = 0$	$p = q = 0.001$
Knocked out norm	Mean (S.D.)	Mean (S.D.)
BBBB [ALLB]	0.816 (0.354)	0.745 (0.399)
BBBG	0.980 (0.008)	0.979 (0.007)
BBGB	0.978 (0.012)	0.979 (0.007)
BBGG	0.923 (0.226)	0.961 (0.134)
BGBB	0.920 (0.225)	0.922 (0.225)
BGBG	0.982 (0.008)	0.977 (0.006)
BGGB	0.959 (0.134)	0.978 (0.007)
BGGG	0.979 (0.011)	0.959 (0.134)
GBBB [SH]	0.025 (0.004)	0.026 (0.005)
GBBG [SJ]	0.120 (0.287)	0.616 (0.457)
GBGB	0.982 (0.007)	0.977 (0.006)
GBGG	0.941 (0.188)	0.978 (0.006)
GGBB [IS]	0.023 (0.006)	0.022 (0.004)
GGBG [ST]	0.412 (0.432)	0.055 (0.060)
GGGB	0.915 (0.225)	0.961 (0.010)
GGGG [ALLG]	0.897 (0.179)	0.371 (0.431)
Without knockout	0.939 (0.187)	0.980 (0.006)

Table S5. The number of transitions of norms with the greatest populations with no error after a cooperation ratio exceeds 0.9 until the end of generations. The simulation runs 50 replications. The transition is counted when the most majority norm is superseded by other norms. Parameters: $b = 5$, $c = 1$, $N = 500$, $R = 500$, $G = 1000$, $p = 0$, $q = 0$. For example, the transition from ALLG to GGGB occurs 929 times. During this time, alternation of norms with the greatest populations for a total of 2,324 times could be observed.

From	To	No.
ALLG	GGGB	929
GGGB	ALLG	920
ST	ALLG	225
ALLG	ST	185
SJ	ST	35
ST	SJ	6
IS	GGGB	3
SH	ALLB	3
ALLB	SH	3
ALLG	IS	3
IS	ST	3
ST	IS	2
GGGB	ST	2
GGGB	SH	1
SH	SJ	1
GGGB	IS	1
ST	GGGB	1
IS	ALLG	1

Table S6. The number of transitions of norms with the greatest populations with errors after cooperation ratio exceeds 0.9 until the end of generations. The setting of the table is the same as Table S5. Parameters: $b = 5$, $c = 1$, $N = 500$, $R = 500$, $G = 1000$, $p = 0.001$, $q = 0.001$.

From	To	No.
GGGB	ALLG	968
ALLG	GGGB	950
ST	ALLG	434
ALLG	ST	426
GGGB	IS	41
IS	GGGB	40
ST	GGGB	15
GGGB	ST	14
IS	ST	12
ALLG	IS	10
ST	IS	9
IS	ALLG	8

Figure

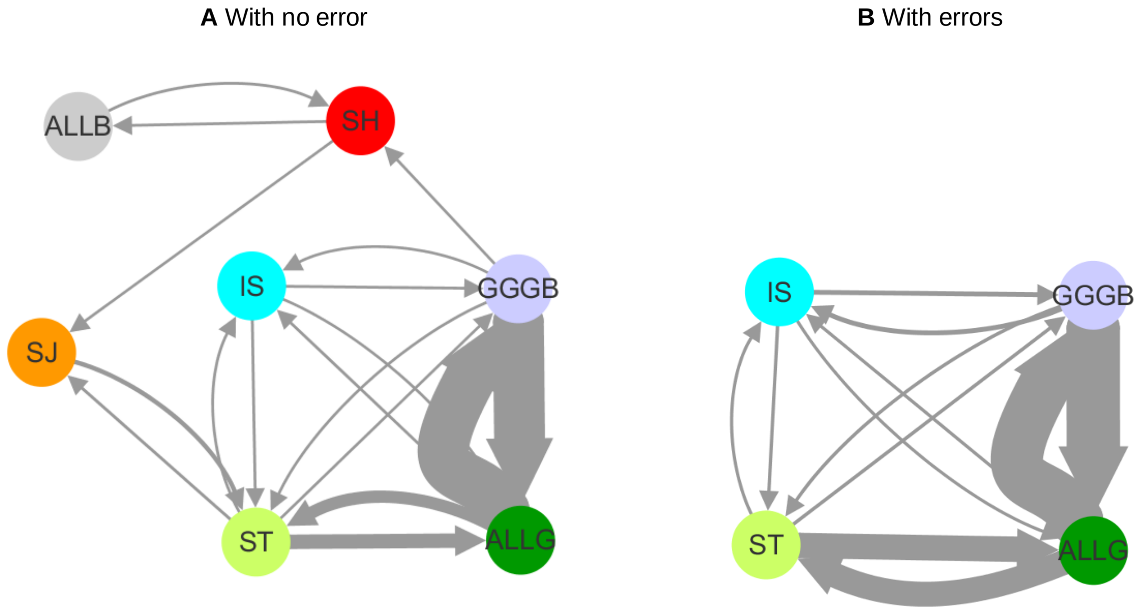

Figure S1. The transition diagram of norms with the greatest populations with no error (A) and with errors (B) after cooperation ratio exceeds 0.9 until the end of generations. Panel A is drawn using the data of Table S5 and panel B is drawn using the data of Table S6. Both panels show that the tolerant norms (such as ALLG, GGGB, and ST) coexist as the majority.
